# Thou Atomy Thou!

**Published:** 1843-12

**Authors:** 


### "THOU ATOMY THOU!"

A late number of the *Reperterio Medico-Habanero*, gives a brief account of an *anatomical man*, named Claudius Ambrose Seurat, five feet three inches in height, and weighing only *forty-three pounds!* He is a native of Troyes, France, and was born in 1798, of healthy parents. The emaciation began at the age of four years, and continued until the twenty-fourth year; it cannot be attributed to sickness, as during this period his health was good. The chest is so much depressed that the sternum is only three inches from the vertebral column. The heart and lungs are situated lower than natural. Respiration is easy; the pulse beats about fifty to the minute, and is feeble. The arms are two inches and a half in circumference, the legs one in thickness. He consumes, daily, about twelve ounces of food, consisting of fish, light meats, &c.; when eating he supports himself with the elbows on the table or on his knees, his extreme debility not permitting him to raise food to the mouth in any other manner. He requires assistance in walking, and lifts the feet very high through fear of falling or stumbling. The intellectual powers are not enfeebled like the physical. He can read and write, and his responses to questions propounded to him exhibit a good discernment, and a correct judgement.

Accompanying the description, are three full length drawings on stone, giving a front, side, and back view of the individual. These, although we are told his aspect is not disagreeable, certainly represent him as a doleful looking object—some such being as must have been Shakspeare's beadle, whom *Doll Tear-sheet* calls "a tripe-visaged rascal"—a "good-man death! good-man bones!"

The only case approaching this, and of which we have any knowledge, is that of Calvin Edson, who weighed *fifty-six* pounds—the height we do not remember. C.

